# Subgroups and Features of Poor Responders to Anti-Vascular Endothelial Growth Factor Treatment in Eyes with Neovascular Age-Related Macular Degeneration

**DOI:** 10.4274/tjo.galenos.2020.38488

**Published:** 2020-10-30

**Authors:** Mine Esen Barış, Jale Menteş, Filiz Afrashi, Serhad Nalçaçı, Cezmi Akkın

**Affiliations:** 1 Ege University Faculty of Medicine, Department of Ophthalmology, İzmir, Turkey

**Keywords:** Anti-vascular endothelial growth factor, neovascular age-related macular degeneration, treatment response

## Abstract

**Objectives:**

This study aimed to determine the incidence of poor response to intravitreal (IV) anti-VEGF treatment in neovascular age-related macular degeneration (nvAMD) and to define subgroups of poor responders.

**Materials and Methods:**

A total of 235 treatment-naive eyes of 202 patients completed this prospective study. Patients younger than 50 years of age and those with a contraindication for anti-VEGF therapy were excluded. All eyes were treated with IV ranibizumab. Poor response was defined as recurrence, persistence, or worsening despite treatment. Poor responders were classified into subgroups based on progression patterns.

**Results:**

Of the 235 eyes, 78 (33.2%) showed poor response. Pigment epithelial detachment (PED) and occult choroidal neovascularization (CNV) were more common among poor responders (p<0.001) and 5 subgroups were identified.

**Conclusion:**

Poor response to anti-VEGF treatment is not uncommon and occult CNV and PED are frequently seen in these eyes. Various subgroups can be defined based on clinical features.

## Introduction

Intravitreal (IV) injection of anti-vascular endothelial growth factors (anti-VEGF) is accepted as a standard treatment method for neovascular age-related macular degeneration (nvAMD).

Of the many multicenteric clinical trials, MARINA (Minimally Classic/Occult Trial of Anti-VEGF Antibody Ranibizumab in the Treatment of Neovascular Age-Related Macular Degeneration) and ANCHOR (Anti-VEGF Antibody for the Treatment of Predominantly Classic Choroidal Neovascularization in AMD) were seminal trials of monthly ranibizumab (Lucentis; Genentech, San Francisco, CA, USA) therapy in eyes with minimally classic and occult nvAMD and predominantly classic nvAMD, respectively. While the 2-year results of these trials demonstrated improved or preserved visual acuity in approximately 90-95% of treated eyes compared to control eyes, vision loss of at least 15 letters (3 lines) despite continued monthly anti-VEGF therapy was also reported in 5-10% of eyes.^1,2,3,4,5^ It has also been noted that eyes showing inadequate or no treatment response and persistent disease activity are those with better baseline visual acuity compared to the group with the greatest letter gains.^6,7^

Identifying eyes with good or poor anatomic response to anti-VEGF drugs, distinguishing different subgroups if present, and knowing the baseline lesion characteristics of eyes with nvAMD are believed to be important for predicting treatment outcomes and determining the causes of resistance.

Therefore, the aim of this prospective clinical trial was to characterize responses to anti-VEGF therapy with ranibizumab in eyes with active nvAMD, to analyze subgroups within the good and poor response groups, and to evaluate their baseline clinical features.

## Materials and Methods

This prospective cohort study included 297 eyes of 245 consecutive patients diagnosed with active nvAMD and treated with IV anti-VEGF therapy in the Retina Unit of the Ege University Medical Faculty Department of Ophthalmology.

Patients less than 50 years of age, those who had previously been treated for nvAMD, those with a contraindication for anti-VEGF therapy or developed complications that might alter the Optical Coherence Tomography (OCT) parameters during treatment, and those who did not follow the treatment protocol were excluded from the study. As a result, 235 eyes of 202 patients completed the study and were included in the evaluation.

An informed voluntary consent form was obtained from each patient, ethical board approval was obtained from the Ege University Clinical Research Ethics Committee (decision no. 12-2/47, 2013) and the Ministry of Health Turkish Pharmaceuticals and Medical Devices Agency (transaction no. 1135321/06.03.2013). The study was conducted in adherence to the principles of the Declaration of Helsinki.

All patients underwent a complete ophthalmologic examination, including best corrected visual acuity (BCVA) determined by Snellen chart, intraocular pressure (IOP) measurement, and biomicroscopic examination of the anterior and posterior segments. Prior to treatment, each patient underwent a spectral domain optic coherence tomography (SD-OCT) scan with a Topcon SD-OCT (Topcon Medical Systems, Paramus, NJ, USA) and Heidelberg Spectralis HRA + OCT (Heidelberg Engineering, Heidelberg, Germany) device, in addition to fluorescein angiography (FA) with a Topcon TRC.50IX device (Topcon Medical Systems, Paramus, USA). Neovascularization (nv) type was assessed based on the presence, type and location of increased central retinal thickness (CRT), subretinal fluid (SRF), intraretinal cysts (IRC), and pigment epithelial detachment (PED) on SD-OCT. CRT evaluations were made based on irregularities in retinal thickness in the central 6x6 mm^2^ area at the posterior pole. The types of nv based on the staining properties of the lesions, as well as dye leakage in late phases, were recorded with FA. Well-demarcated areas of intense hyperfluorescence appearing early and showing progressive leakage were accepted as classical choroidal neovascularization (CNV), whereas fibrovascular PEDs and late leakage of undetermined source were evaluated as occult CNV. In case of mixed types, the lesion was considered predominantly classical if more than 50% consisted of classical component and minimally classical if it comprised 1-50% classical component. Types of nv based on location on SD-OCT images were also noted as type 1 (sub-retinal pigment epithelium [RPE]), type 2 (subretinal), and type 3 (intraretinal).

Eyes exhibiting fresh hemorrhage in clinical examination, findings of SRF, IRC, or sub-RPE fluid on SD-OCT, and leakage on FA were classified as having active nvAMD. These eyes were treated with IV ranibizumab (0.5 mg/0.05 mL ranibizumab, Lucentis; Genentech Inc., San Francisco, CA, USA) under fully sterile operating room conditions.

Follow-up examinations were performed 4-6 weeks after treatment. BCVA and SD-OCT findings were reevaluated and IV ranibizumab injections were repeated for eyes with signs of persistent activity (fresh hemorrhage, SRF, IRC, or sub-RPE fluid).

**Figure 1:**
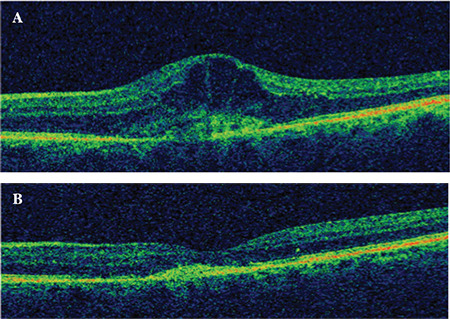
Spectral domain optical coherence tomography (SD-OCT) images of an eye that showed good response to intravitreal anti-VEGF injections. A) Initial image shows intraretinal cysts and increased central retinal thickness. B) SD-OCT image after 6 consecutive injections

Eyes that showed full regression or resorption in follow-up examinations before or after completing the 6 injections were classified as “good responders” (Figure 1), while eyes with recurrence, persistence, or progressive worsening after 6 injections were classified as “poor responders”. Visual acuity was not considered as a parameter in our definitions of response or poor response. The differences in baseline features between eyes in the two groups were statistically analyzed. Treatment was stopped in eyes that showed total regression of activation signs before completing 6 injections and these eyes were considered good responders. These patients were seen in regular follow-up visits and injections started again if they showed any sign of activation. Patients who still needed anti-VEGF treatment after 6 injections continued to receive treatment as long as they needed.

**Figure 2:**
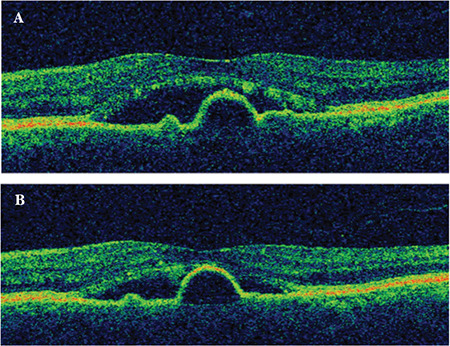
An eye with partial nonresponse to treatment A) before treatment and B) after 6 injections. There was only a minimal change in spectral domain optical coherence tomography findings despite treatment

**Figure 3:**
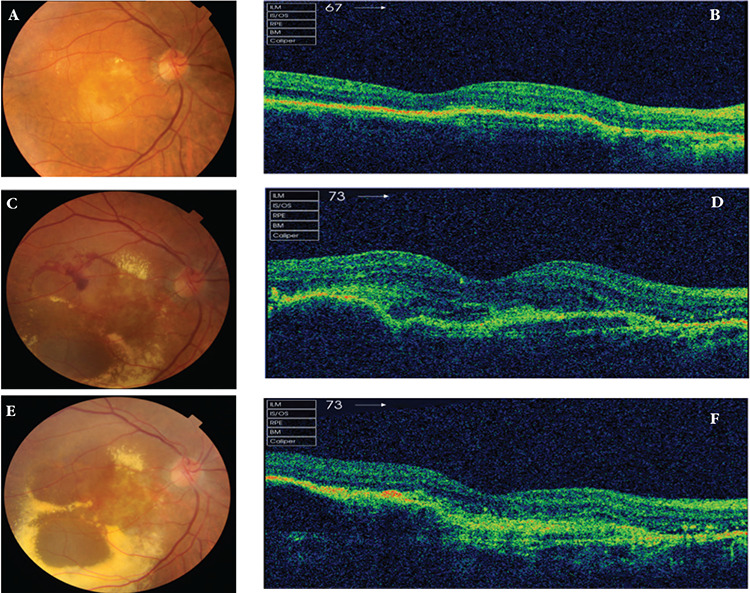
An example from the “worsening” subgroup. A) Fundus photograph and B) spectral domain optical coherence tomography image at the time of initial examination. C and D) Images obtained after 6 injections show increased central retinal thickness, subretinal fluid, increased exudation, and a fresh hemorrhage. E and F) After 9 injections, there is a marked increase in exudation

**Figure 4:**
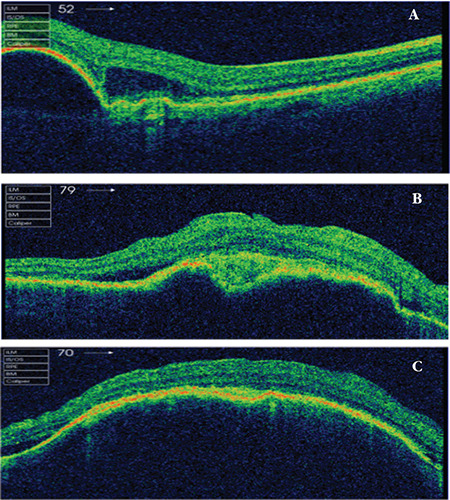
Spectral domain optical coherence tomography images of an eye in the “non-response over time” subgroup. A) Before treatment. B), Pigment epithelial detachment (PED) height and subretinal fluid amount were reduced after 6 injections. C) Images obtained after 9 injections show the PED returned to pretreatment height despite ongoing treatment

Poor responders were divided into 5 subgroups by analyzing anatomical findings and response characteristics:

1. True nonresponders: Eyes with no change in signs of activity (SRF, IRF, sub-RPE fluid, fresh hemorrhage) during treatment;

2. Partial nonresponders: Eyes exhibiting partial improvement (e.g., minimal regression in SRF and/or IRF) in signs of activity during treatment (Figure 2);

3. Anti-VEGF dependents: Eyes that showed complete regression of signs of activity with treatment but were unable to tolerate intervals longer than 4-6 weeks between injections without showing recurrence (increase in SRF/IRF, sub-RPE fluid or PED size);

4. Worsening: Eyes with progression of anatomic findings, with exudate or hemorrhage, despite treatment (Figure 3);

5. Nonresponse over time: Eyes that initially responded well to treatment but became unresponsive over time due to reduction in drug effectiveness with continued treatment (Figure 4).

### Statistical Analysis

SPSS 15.0 package software was used for statistical analyses. Independent samples t-test, chi-square test, and Fisher’s Exact test were used to evaluate the findings, with p values <0.05 were accepted as statistically significant.

## Results

Of the 202 patients, 102 (50.5%) were male and 100 (49.5%) were female; 33 (16.3%) had bilateral nvAMD, and the mean age was 74.03±7.8 (56-89) years.

Of the 235 eyes, treatment response to anti-VEGF therapy with IV ranibizumab was evaluated as good in 157 eyes (66.8%) and poor in 78 eyes (33.2%). Of the 33 bilateral patients, 17 showed good response and 7 showed poor response to treatment, while 9 patients had 1 eye in each group.

The demographic characteristics, lens status, pre- and post- treatment BCVA, number of injections, and follow-up periods pertaining to the eyes with good and poor treatment responses are shown in Table 1. There were no statistically significant differences between the groups in terms of age and gender distribution (p=0.22 and p=0.48, respectively; t-test and chi-square test). The groups were also statistically comparable in terms of lens status (pseudophakic or phakic) (p=0.8; Fisher’s Exact test). Comparison of BCVA between the groups revealed no statistically significant differences either pre- or posttreatment (p=0.38 and p=0.06 respectively; t-test and Fisher’s Exact test). Eyes with poor treatment response had significantly higher mean number of injections and longer follow-up period compared to eyes with good response (p<0.001 and p<0.001; t-test).

Twenty-one eyes (26.9%) were categorized as true nonresponders, 29 eyes (37.2%) as partial nonresponders, 13 eyes (16.7%) as anti-VEGF dependents, 11 eyes (14.1%) as worsening, and 4 eyes (5.1%) as showing nonresponse over time.

The baseline SD-OCT and FA features of the eyes in both groups are shown in Table 2. The number of eyes with increased CRT and IRC in the good responders group was significantly higher compared to the poor responders group, while there was no significant difference in terms of SRF (p=0.02, p=0.004, p=0.4; Fisher’s Exact test). Absence of PED was significantly more common among good responder eyes compared to poor responders (p<0.001; chi-square test). Poor responder eyes had an initial PED rate of 88.5% and a significantly higher prevalence of fibrovascular PED (77%) compared to good responders (39.5%) (p<0.001; chi-square test). Comparison of the nv types based on SD-OCT location between the two groups showed that type 2 nv (subretinal) was significantly more common in good responders, while type 1 nv (sub-RPE) was significantly more common in poor responders (p=0.03 and p=0.04, respectively; chi-square test).

In terms of baseline lesion characteristics on FA, predominantly classic nv (53.5%) was significantly more common in the good responders group, while occult nv (70.5%) was significantly more common among poor responders (p<0.001 for both; chi-square test).

The baseline SD-OCT and FA features of the poor responder subgroups are shown in Table 3. There was no statistically significant difference between the subgroups in terms of increased CRT or presence of SRF, IRC, or presence and type of PED (p=0.82, p=0.78, p=0.62, and p=0.94, respectively; chi-square test). There was also no difference between the subgroups in terms of the nv types identified via SD-OCT and FA (p=0.33; chi-square test).

## Discussion

In this prospective clinical trial, 235 eyes with nvAMD received consecutive doses of IV ranibizumab therapy at intervals of 4-6 weeks, and treatment response was defined as good in 157 eyes (66.8%) and poor in 78 eyes (33.2%). Criteria for poor response in this trial included persistent, recurrent, or progressive signs of nvAMD activity in clinical examination or SD-OCT performed 1 month after 6 doses of IV ranibizumab.

Although IV injection of anti-VEGF agents is currently accepted as a standard treatment method for active nvAMD, the rate of unresponsiveness to treatment reported in different trials varies widely (7.5-68.1%).^8,9^ The main reason for these differences is the use of different criteria when assessing treatment response. There is still no consensus among clinicians as to whether regression of signs of activity or improvement in visual acuity should be accepted as the primary criterion of treatment response, or after how many doses response should be evaluated.^10^ Treatment response was defined according to changes in BCVA in the MARINA and ANCHOR trials, which were the first trials to demonstrate the efficacy of ranibizumab. In these trials, preserved or improved (15 letters or more) BCVA was reported for 90% of the patients who received monthly anti-VEGF therapy for 24 months, and losses of more than 15 letters were reported for the other 10% of patients. In clinical practice, however, there are few studies in which BCVA is accepted as the treatment response criterion.^9,11,12,13^ Most clinicians evaluate response and decide to repeat treatment based on signs of activity detected on examination and SD-OCT (in other words, based on anatomic findings rather than an increase in BCVA), with regression or complete resolution of these findings considered good treatment response.^8,14,15,16,17,18,19,20,21,22,23,24,25,26,27,28^

In a retrospective study involving 218 eyes, Otsuji et al.^13^ considered eyes with no increase in BCVA and/or no reduction in CRT despite 3 consecutive doses of IV ranibizumab therapy administered at 4-week intervals as unresponsive to treatment, reporting the rate of unresponsiveness as 10.1%. Shin et al.^8^ retrospectively evaluated 267 nvAMD cases and determined that 7.5% were unresponsive to anti-VEGF therapy (ranibizumab and bevacizumab). In their study, persistent and/or increased intraretinal or subretinal exudate despite 3 consecutive IV injections was accepted as the criterion for unresponsiveness. Byun et al.^9^ analyzed treatment response in 113 consecutive eyes with nvAMD that received IV bevacizumab injections for 1 year, describing eyes that showed less than 7-11 ETDRS letters improvement in BCVA as unresponsive (68.1%).

Slakter^14^ suggested that BCVA may not increase and may even decrease despite complete regression of signs of activity and remission of disease in good responders to anti-VEGF therapy. He attributed this to changes that occur secondarily to nvAMD such as subretinal fibrosis and scar formation or RPE and photoreceptor atrophy, stating that for these reasons BCVA is not a reliable criterion for determining responsiveness or unresponsiveness to treatment. In the present study, we used the regression of signs of nvAMD activity to define response to ranibizumab therapy. There were no statistically significant differences in BCVA between good and poor responders in our study. As indicated by Slakter, we believe this is due to secondary changes that occurred in some eyes that showed good treatment response.

There is also no consensus regarding when to evaluate treatment response among clinical trials. Assessments were done after 3 or 6 consecutive injections in the vast majority of trials^8,9,13,26^ while in some trials this number is reported as 9, 12, or more.^23^ In the present trial, the eyes were re-evaluated 1 month after receiving the last of 6 consecutive ranibizumab injections. Eyes showing a poor response received a significantly higher mean number of injections and had a significantly longer mean follow-up period compared to eyes with good treatment response.

In our study, the prevalence of predominantly classic nv was higher among good responders, while occult nv was more common among poor responders, and this difference was found to be statistically significant. Previously published studies have differed on this point. Lux et al.^12^ detected no difference in nv type between responsive and unresponsive eyes, but reported that the unresponsive group had significantly larger baseline nv area. Otsuji et al.^13^ determined that occult nv was more prevalent than classic nv among poor responders, whereas response/nonresponse was not associated with baseline nv dimensions. Hörster et al.^29^ reported that predominantly classic and minimally classic nv required more injections than occult nv. However, these data have not been supported by the results of other studies. Veritti et al.^30^ stated that less satisfactory outcomes were achieved when treating eyes with occult nv associated with nvAMD compared to other types of nv.

In our study, increased CRT and presence of IRC were significantly more common among good responders compared to poor responders, while the groups showed no difference in terms of SRF presence. Shin et al.^8^ divided non-responders into two groups those who had SRF only and those who had predominantly IRC and found that the eyes with SRF were less responsive to treatment compared to eyes with IRC. Guber et al.^31^ also reported that eyes with IRC responded better to treatment than those with SRF or PED and showed a more pronounced reduction in CRT. Tannan et al.^32^ reported that pretreatment SRF was associated with longer duration of anti-VEGF therapy.

In our study, there was a significant difference between good and poor responder eyes in terms of baseline PED presence (47.1% and 88.5%, respectively). In addition, the prevalence of fibrovascular PED was significantly higher in poor responders (77%) compared to good responders (39.5%). Inoue et al.^33^ observed a greater BCVA improvement in eyes with baseline serous PED compared to eyes with fibrovascular PED. Punjabi et al.^34^ categorized PEDs as empty, solid, or mixed based on their appearance on OCT, reporting the rate of complete or partial regression with treatment to be 3% for solid PEDs and 46% for empty PEDs.

Our evaluation of poor responders to anti-VEGF therapy with ranibizumab based on clinical response and SD-OCT findings revealed 5 distinct subgroups. The most common pattern was partial non-response (37.2%), which was characterized by partial improvement in signs of activity during treatment. Furthermore, some eyes responded well to treatment but required another injection every month and could not tolerate treatment intervals longer than 4-6 weeks. These eyes were referred to as “anti-VEGF dependent” (16.7%). Approximately 5% of the eyes showed good initial response but became unresponsive due to diminished effect of the drug over time, and these were classified in the “non-response over time” group. Publications on tachyphylaxis, defined as a reduction in the effectiveness of a drug on tissue after repeated administration, have reported that this phenomenon occurs after at least 5 consecutive anti-VEGF injections, with an incidence of 2%.^21^ A search of the literature did not yield any studies on the development of tolerance to anti-VEGF drugs.

In his 2010 review, Slakter^14^ stated that there are many patients who do not exhibit the desired response to ranibizumab therapy and whose exudative findings persist or progress; he referred to these patients as “anti-VEGF nonresponders” and described 5 subgroups within this group. The article does not provide data on the prevalence and baseline clinical features of the subgroups, but 3 of the described subgroups are similar to those in our study. To the best of our knowledge, ours is the first clinical study to determine subgroups of poor responders to anti-VEGF therapy and evaluate their prevalence.

## Conclusion

The value of anti-VEGF drugs as effective and safe therapies for the treatment of nvAMD is undisputable. However, poor response or nonresponse to anti-VEGF drugs in some eyes is an important issue in clinical practice. In addition to determining the prevalence of these suboptimal responses in clinical studies, our results suggest that identifying baseline features of these eyes and conducting subgroup analysis will be beneficial in order to investigate the causes of unresponsiveness and to modify and improve treatment strategies in such cases.

## Figures and Tables

**Table 1:**
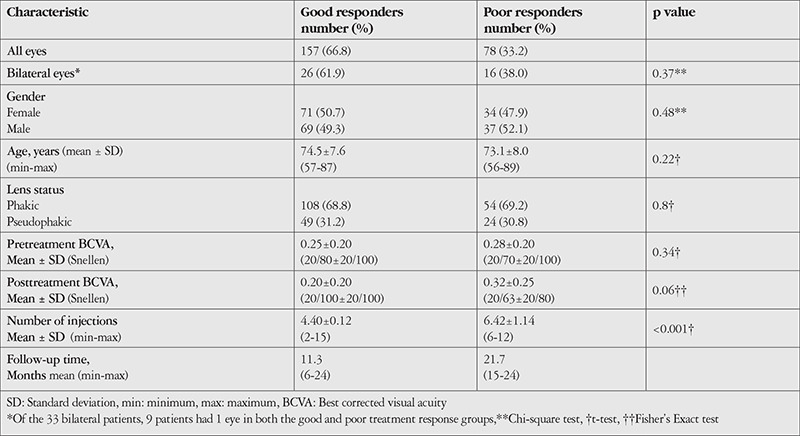
Demographic characteristics, lens status, best corrected visual acuity, number of injections, and follow-up periods of good and poor responder eyes

**Table 2:**
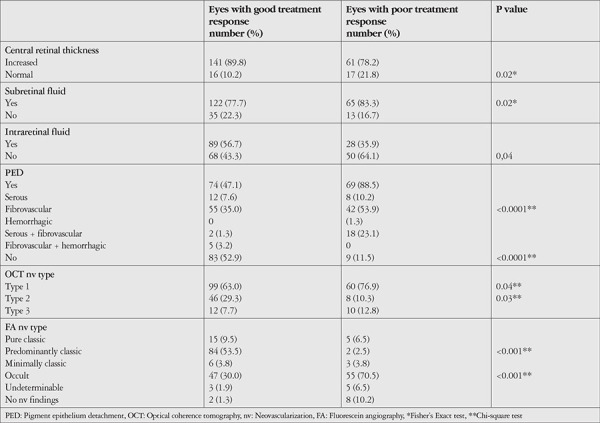
Baseline optical coherence tomography and fluorescein angiography characteristics in the good and poor responder groups

**Table 3:**
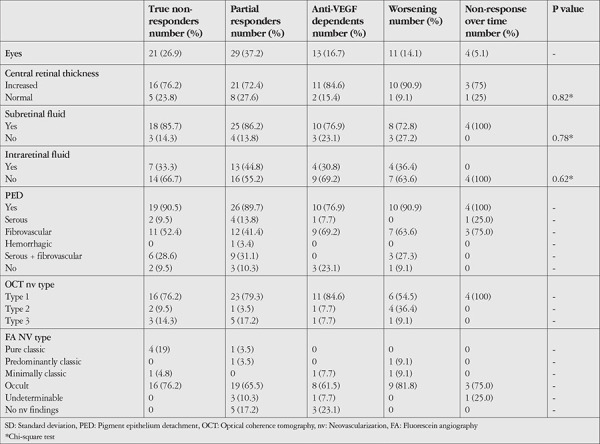
Baseline optical coherence tomography and fluorescein angiography characteristics in subgroups of poor responders

## References

[1] Kaiser PK, Brown DM, Zhang K, Hudson HL, Holz FG, Shapiro H, Schneider S, Acharya NR (2007). Ranibizumab for predominantly classic neovascular age-related macular degeneration: subgroup analysis of first-year ANCHOR results. Am J Ophthalmol..

[2] Brown DM, Michels M, Kaiser PK, Heier JS, Sy JP, Ianchulev T;, ANCHOR Study Group (2009). Ranibizumab versus verteporfin photodynamic therapy for neovascular age-related macular degeneration: two-year results of the ANCHOR study. Ophthalmology..

[3] Bressler NM, Chang TS, Suñer IJ, Fine JT, Dolan CM, Ward J, Ianchulev T;, MARINA and ANCHOR Research Groups (2010). Vision related function after ranibizumab treatment by better- or worse-seeing eye: clinical trial results from MARINA and ANCHOR. Ophthalmology..

[4] Martin DF, Maguire MG, Ying GS, Grunwald JE, Fine SL, Jaffe GJ;, CATT Research Group (2011). Ranibizumab and bevacizumab for neovascular age-related macular degeneration. N Engl J Med..

[5] Mitchell P, Korobelnik JF, Lanzetta P, Holz FG, Prünte C, Schmidt-Erfurth U, Tano Y, Wolf S (2010). Ranibizumab (lucentis) in neovascular age-related macular degeneration: evidence from clinical trials. Br J Ophthalmol..

[6] Rosenfeld PJ, Shapiro H, Tuomi L, Webster M, Elledge J, Blodi B;, MARINA and ANCHOR Study Groups (2011). Characteristics of patients losing vision after 2 years of monthly dosing in the phase III ranibizumab clinical trials. Ophthalmology..

[7] Mariani A, Deli A, Ambresin A, Mantel I (2011). Characteristics of eyes with secondary loss of visual acuity receiving variable dosing ranibizumab for neovascular age-related macular degeneration. Graefes Arch Clin Exp Ophthalmol..

[8] Shin JY, Woo SJ, Ahn J, Park KH (2013). Anti-VEGF-refractory exudative age-related macular degeneration: differential response according to features on optical coherence tomography. Korean J Ophthalmol..

[9] Byun YJ, Lee SJ, Koh HJ (2010). Predictors of response after intravitreal bevacizumab injection for neovascular age-related macular degeneration. Jpn J Ophthalmol..

[10] Amoaku WM, Chakravarthy U, Gale R, Gavin M, Ghanchi F, Gibson J, Harding S, Johnston RL, Kelly SP, Lotery A, Mahmood S, Menon G, Sivaprasad S, Talks J, Tufail A, Yang Y (2015). Defining response to anti-VEGF therapies in neovascular AMD. Eye (London)..

[11] Cohen SY, Mimoun G, Oubraham H, Zourdani A, Malbrel C, Queré S, Schneider V;, LUMIERE Study Group (2013). Changes in visual acuity in patients with wet age-related macular degeneration treated with intravitreal ranibizumab in daily clinical practice: the LUMIERE study. Retina..

[12] Lux A, Llacer H, Heussen FM, Joussen AM (2007). Non-responders to bevacizumab (Avastin) therapy of choroidal neovascular lesions. Br J Ophthalmol..

[13] Otsuji T, Nagai Y, Sho K, Tsumura A, Koike N, Tsuda M, Nishimura T, Takahashi K (2013). Initial non-responders to ranibizumab in the treatment of age-related macular degeneration (AMD). Clin Ophthalmol..

[14] Slakter JS (2010.). What to do when anti-VEGF therapy fails. Retin Physician.

[15] Keane PA, Liakopoulos S, Ongchin SC, Heussen FM, Msutta S, Chang KT, Walsh AC, Sadda SR (2008). Quantitative subanalysis of optical coherence tomography after treatment with ranibizumab for neovascular age-related macular degeneration. Invest Ophthalmol Vis Sci..

[16] Ying GS, Huang J, Maguire MG, Grunwald JE, Jaffe GJ, Martin DF;, CATT Research Group (2012). Baseline predictors of visual acuity response to ranibizumab and bevacizumab in the comparison of AMD treatment trial (CATT). Invest Ophthalmol Vis Sci..

[17] Binder S (2012). Loss of reactivity in intravitreal anti-VEGF therapy: tachyphylaxis or tolerance?. Br J Ophthalmol.

[18] Gasperini JL, Fawzi AA, Khondkaryan A, Lam L, Chong LP, Eliott D, Walsh AC, Hwang J, Sadda SR (2012). Bevacizumab and ranibizumab tachyphylaxis in the treatment of choroidal neovascularisation. Br J Ophthalmol..

[19] Lalwani GA, Rosenfeld PJ, Fung AE, Dubovy SR, Michels S, Feuer W, Davis JL, Flynn HW Jr, Esquiabro M (2009). A Variable-dosing regimen with ıntravitreal ranibizumab for neovascular age-related macular degeneration: year 2 of the PrONTO study. Am J Ophthalmol..

[20] Jang L, Gianniou C, Ambresin A, Mantel I (2015). Refractory subretinal fluid in patients with neovascular age related macular degeneration treated with intravitreal ranibizumab: visual acuity outcome. Graefes Arch Clin Exp Ophthalmol.

[21] Eghoj MS, Sorensen TL (2012). Tachyphylaxis during treatment of exudative age-related macular degeneration with ranibizumab. Br J Ophthalmol..

[22] Kovach JL, Schwartz SG, Flynn HW Jr, Scott IU (2012). Anti-VEGF treatment strategies for wet AMD. J Ophthalmol..

[23] Krebs I, Glittenberg C, Ansari-Shahrezaei S, Hagen S, Steiner I, Binder S (2013). Non-responders to treatment with antagonists of vascular endothelial growth factor in age related macular degeneration. Br J Ophthalmol..

[24] Wang VM, Rosen RB, Meyerle CB, Kurup SK, Ardeljan D, Agron E, Tai K, Pomykala M, Chew EY, Chan CC, Tuo J (2012). Suggestive association between PLA2G12A single nucleotide polymorphism rs2285714 and response to anti-vascular endothelial growth factor therapy in patients with exudative age related macular degeneration. Mol Vis..

[25] Wykoff CC, Clark WL, Nielsen JS, Brill JV, Greene LS, Heggen CL (2018). Optimizing anti-VEGF treatment outcomes for patients with neovascular age related macular degeneration. J Manag Care Spec Pharm.

[26] Rich RM, Rosenfeld PJ, Puliafito CA, Dubovy SR, Davis JL, Flynn HW Jr, Gonzalez S, Feuer WJ, Lin RC, Lalwani GA, Nguyen JK, Kumar G (2006). Shortterm safety and efficacy of intravitreal bevacizumab (Avastin) for neovascular age-related macular degeneration. Retina..

[27] Schaal S, Kaplan HJ, Tezel TH (2008). Is there tachyphylaxis to intravitreal antivascular endothelial growth factor pharmacotherapy in age-related macular degeneration?. Ophthalmology.

[28] Forooghian F, Cukras C, Meyerle CB, Chew EY, Wong WT (2009). Tachyphylaxis after intravitreal bevacizumab for exudative age-related macular degeneration. Retina..

[29] Hörster R, Ristau T, Sadda SR, Liakopoulos S (2011). Individual recurrence intervals after anti-VEGF therapy for age-related macular degeneration. Graefes Arch Clin Exp Ophthalmol..

[30] Veritti D, Macor S, Menchini F, Lanzetta P (2013). Effects of VEGF inhibition on retinal morphology, neovascular network size, and visual acuity in patients with vascularized pigment epithelium detachment because of occult choroidal neovascularization. Retina..

[31] Guber J, Henrich PB, Guber I, Cybulska A, Flammer J, Josifova T (2012). Predictive factors for poor central retinal thickness response to ranibizumab in wet AMD. Invest Ophthalmol Vis Sci..

[32] Tannan A, Li C, Stevens TS (2010). Predicting treatment outcomes in patients with newly diagnosed exudative age-related macular degeneration based on initial presentation. Invest Ophthalmol Vis Sci..

[33] Inoue M, Arakawa A, Yamane S, Kadonosone K (2013). Variable response of vascularized pigment epithelial detachments to ranibizumab based on lesion subtypes, including polypoidal choroidal vasculopathy. Retina..

[34] Punjabi OS, Huang J, Rodriguez L, Lyon AT, Jampol LM, Mirza RG (2013). Imaging characteristics of neovascular pigment epithelial detachments and their response to anti-vascular endothelial growth factor therapy. Br J Ophthalmol..

